# *Silene sunhangii* (Caryophyllaceae), a new species from China

**DOI:** 10.3897/phytokeys.135.36426

**Published:** 2019-12-05

**Authors:** Nan Lin, Dai-Gui Zhang, Xian-Han Huang, Jian-Wen Zhang, Jing-Yuan Yang, Komiljon Tojibaev, Heng-Chang Wang, Tao Deng

**Affiliations:** 1 CAS Key Laboratory for Plant Diversity and Biogeography of East Asia, Kunming Institute of Botany, Chinese Academy of Sciences, Kunming, Yunnan 650201, China Kunming Institute of Botany, Chinese Academy of Sciences Kunming China; 2 CAS Key Laboratory of Plant Germplasm Enhancement and Specialty Agriculture, Wuhan Botanical Garden, Chinese Academy of Sciences, Wuhan, Hubei 430074, China Wuhan Botanical Garden, Chinese Academy of Sciences Wuhan China; 3 University of Chinese Academy of Sciences, Beijing 100049, China University of Chinese Academy of Sciences Beijing China; 4 Key Laboratory of Plant Resources Conservation and Utilization, Jishou University, Jishou, Hunan 416000, China Jishou University Jishou China; 5 Administration of Shennongjia National Park, Shennongjia, Hubei 44241, China Administration of Shennongjia National Park Shennongjia China; 6 Central Herbarium of Uzbekistan, Institute of Botany, Academy Sciences of Uzbekistan, Tashkent 100025, Uzbekistan Institute of Botany, Academy Sciences of Uzbekistan Tashkent Uzbekistan

**Keywords:** *
Silene
*, new species, morphology, phylogeny, China

## Abstract

*Silene sunhangii*, a new species of Caryophyllaceae known from only three populations in Hubei and Hunan provinces of central China, is described. Both morphological and molecular data were used to assess the taxonomic status and relationships of this species. Morphologically, *S. sunhangii* is most similar to *S. platyphylla* Franch. from which it differs most readily in having 3-veined elliptical leaves without pubescence, tasseled catacorolla, pale purple to red petals without a linear lobe or narrow tooth and lanceolate, bifid to one third. A phylogenetic analysis based on nuclear ITS region identified the new species as a well-supported, independent lineage. Our new species is nested within a grade that encompasses species representing a polyphyletic Silene sect. Physolychnis (Benth.) Bocquet. Both the genetic and morphological data support the recognition of *Silene sunhangii* as a distinct species, although there is inconsistency between these two datasets as to the relationships of the new species.

## Introduction

*Silene* L. (Sileneae, Caryophyllaceae) is the largest genus of Caryophyllaceae Juss., containing over 700 species (Melzheimer 1988; Rautenberg et al. 2010; Oxelman et al. 2011). It is distributed mainly in the Northern Hemisphere, but some species also occur in Africa and South America (Oxelman et al. 2011). Morphologically, *Silene* is characterized by having a synsepalous calyx, 3–5 carpels and a campanulate, clavate or ovate calyx tube. de Candolle (1824) had recognized just eight sections, using several morphological features, including those of habit, inflorescences and stems. Using life form as the primary character, Boissier (1867) recognized 31 sections for the genus, 11 containing annual species and 20 containing perennial species. Previously, the sectional classification within *Silene* was subsequently revised by Chowdhuri (1957) who recognized 44 sections, and it is this scheme that remains in place today. That study was based on a comprehensive sampling of species and a re-assessment of morphological characters. Uncertainties exist as to the number of subgenera that should be recognized for the genus. Rohrbach (1868) recognized two subgenera (subg. Silene and subg. Behenantha (Otth) Endl., based on seed characters) while Williams (1896) recognized three subgenera (subg. Gastrosilene Williams, subg. Conosilene Williams and subg. Eusilene Williams, based on calyx characters). Recent molecular studies (Oxelman et al. 1997; Petri and Oxelman 2011) support the subdivision of *Silene* into two major clades which correspond to subg. Silene and subg. Behenantha. Notwithstanding the above, deficiencies still exist within current classifications involving the genus and a comprehensive phylogenetic study is needed, especially as there is a suggestion in the results of both Oxelman et al. (1997) and Petri and Oxelman (2011) that *Silene* may be polyphyletic.

The treatment of *Silene* by Zhou et al. (2001) in the Flora of China recognized 110 species, of which 67 are endemic and geographically restricted within the country. Within China, species of *Silene* are widely distributed and show a large range of morphological variation. Historically, these species have been accommodated in 22 sections that were defined mostly by characters of the stems, petals, calyx and seeds (Zhou et al. 2001).

Field investigations conducted during this study revealed the existence of a distinctive entity of *Silene* in Hubei and Hunan provinces. Morphologically, this entity is most similar to *S. platyphylla* Franch. which occurs in Yunnan, but it differs significantly from that species in the characters of its root, leaves, petals, catacorolla and lobes. These morphological differences are supported by molecular evidence that justify the recognition of the Hubei and Hunan entity as a new species of *Silene* for China. It is therefore described below as *Silene sunhangii*.

## Material and methods

### Morphology

**Figure 1.**
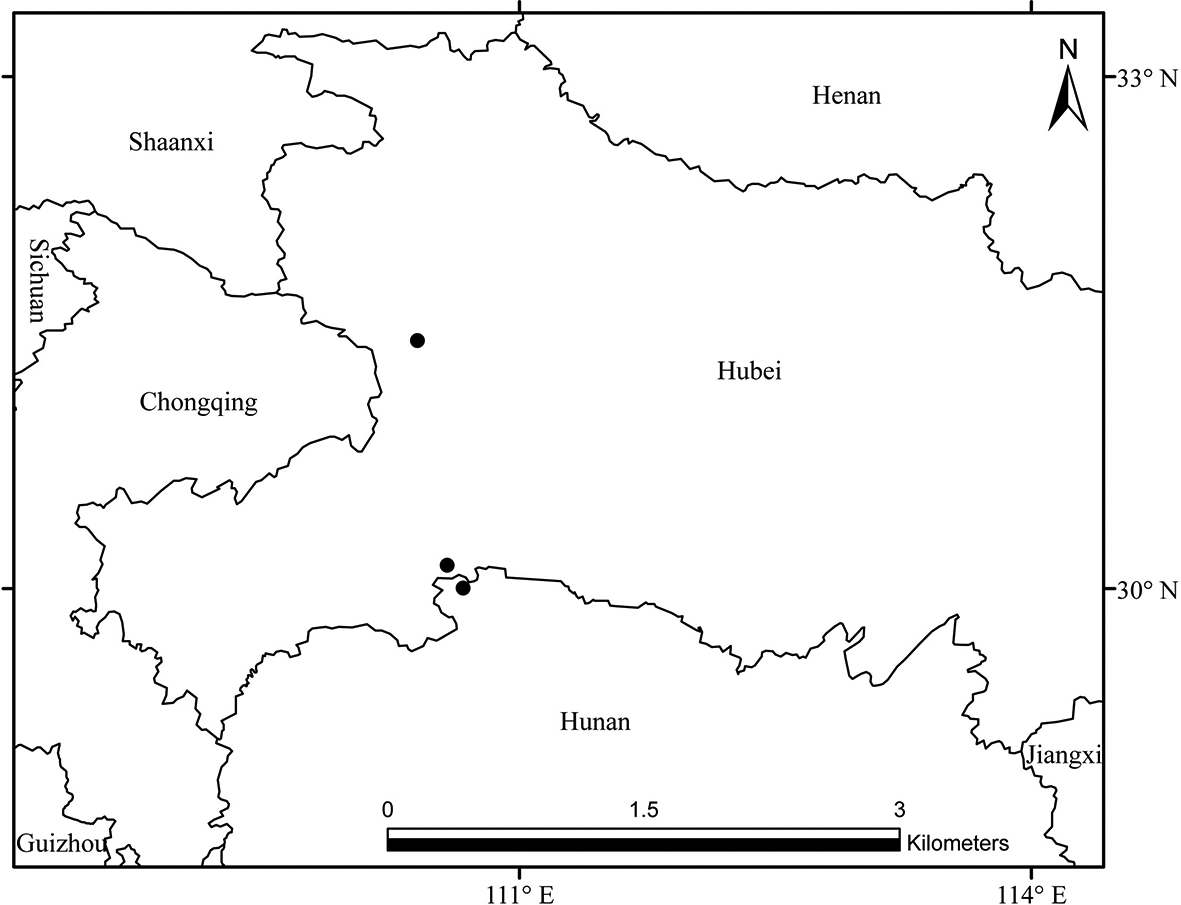
Distribution map of *Silene sunhangii*. The black dots represent locations of *S. sunhangii*.

Natural populations of the new species were collected from three populations in Hubei and Hunan province (Fig. 1, these data were submitted to PANGAEA, accession number 10.1594/PANGAEA.906581). Morphological characters recorded for the new species were based on fresh flowering and fruiting material collected from those populations. *S. platyphylla* were from herbarium material (KUN). A comparison of the new species with similar species is provided in Table 1.

**Table 1.** Comparison of *Silene sunhangii* with similar species detected by morphology (*S. platyphylla*).

Species	Characters	
	*S. sunhangii*	*S. platyphylla*
Roots	tuberous	cylindric
Stems	diffuse, 30–80 cm tall, long pubescent	diffuse, 60–100 cm tall, pubescent
Leaves	elliptic, 4–10 × 1–5 cm, glabrous, conspicuously 3-veined	ovate, 6–8 × 3–5 cm, margin ciliate, 3 or 5-veined
Flower diameter	35–40 mm	20 mm
Pedicel length &indumentum	20–30 mm, pubescent	10–30 mm, hairy
Calyx	tubular-clavate, teeth triangular, glabrous	tubular-clavate, teeth triangular-lanceolate with margin ciliate
Petals	pale purple to red, 2.5 cm, catacorolla tasseled, bifid to one third, lobes lanceolate, without a linear lobe or narrow tooth on each side	white or pale red, 2 cm, catacorolla elliptical or linear, bifid to middle, lobes elliptic, with a linear lobe or narrow tooth on each side
Stamens and filaments	stamens and filaments slightly exserted; filaments pubescent	stamens slightly exserted; filaments glabrous
Distribution	China: Western Hubei and north-western Hunan	China: Western Yunnan

### Molecular analyses

Fresh leaves of the new species were dried in silica gel and total genomic DNA was extracted from 10–20 mg dried leaf tissue. Molecular material of *S. platyphylla* was collected from herbarium specimens (Appendix 1). The nuclear ITS locus was used for phylogeny. The PCR protocol used the following conditions: 5 min at 94, followed by 35 cycles of 1 min at 94 °C, 1 min at 53 °C, 2 min at 72 °C and then ending with a final extension of 5 min at 72 °C. The ITS primers used were ITS1 and ITS4, as described by White et al. (1990) and Urbatsch et al. (2000). Voucher specimen and GenBank accession information for taxa are listed in Appendix 1. DNA sequences were aligned using MAFFT software and then manually checked (Katoh et al. 2002). A total of 301-taxon data sets, including two newly published sequences, were obtained. Bayesian inference (BI) and Maximum likelihood (ML) analyses were conducted using MrBayes 3.1.2 and RAxML v.6 (Huelsenbeck and Ronquist 2001; Stamatakis 2006), respectively. The best-fitting substitution models GTR for Bayesian inference were selected using ModelTest v.3.8, and branch support was computed with 1, 000 bootstrap replicates (Posada and Crandall 1998). ML analyses were conducted using the GTRGAMMA model with 1, 000 nonparametric bootstrapping replicates.

## Results and discussion

### Taxonomic treatment

#### Silene sunhangii

Taxon classificationPlantaeCaryophyllalesCaryophyllaceae

D.G.Zhang, T.Deng & N.Lin sp. nov.

C8593475-D4D5-5648-A09D-ACA031454BB6

urn:lsid:ipni.org:names:77203328-1

Figs 1 , 2 , 3

##### Type.

China. Hubei Province: Shennongjia National Nature Reserve (SNNR) region, Guanmen Mountain, Alt. 1,319 m, 30°08'16.80"N, 110°34'33.59"E, 1 July 2010, Dai-Gui Zhang, et al. 0622 (holotype: KUN!).

##### Diagnosis.

*Silene sunhangii* is morphologically similar to *S. platyphylla*, from which it differs through the root tuberous (not cylindric as *S. platyphylla*), stems 30–80 cm tall (100 cm tall in *S. platyphylla*), leaves elliptic (not obovate in *S. platyphylla*), 3-veined (not 3/5 veined in *S. platyphylla*) and glabrous (not margin ciliate as *S. platyphylla*), flowers 35–40 mm diam. (not 20 mm in *S. platyphylla*), petals purple to red (not white or pale red in *S. platyphylla*), catacorolla tasseled (not elliptic or linear in *S. platyphylla*), lobe limbs divided to 1/3 (more than 1/3 in *S. platyphylla*).

##### Description.

**Figure 2.**
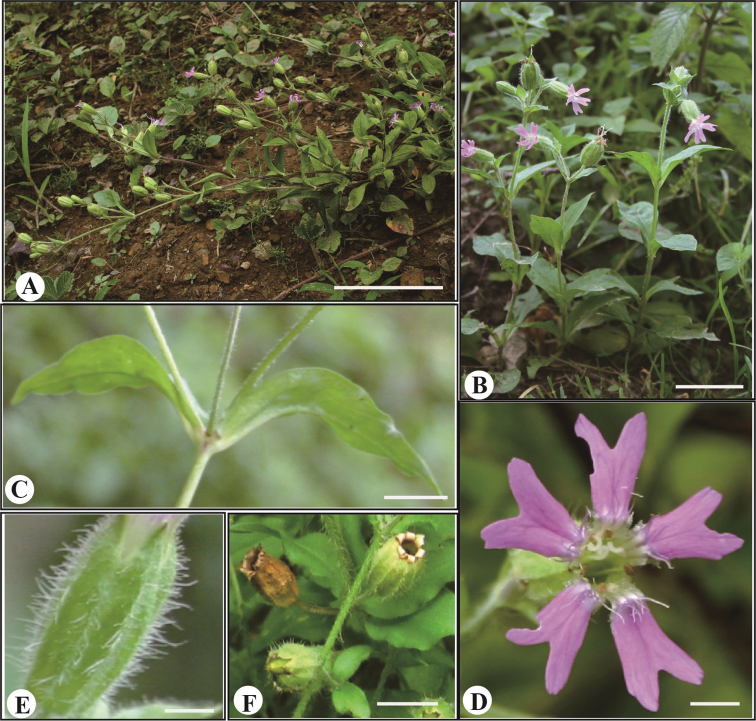
*Silene sunhangii* (from the holotype plant). **A** plant habit **B** plant **C** leaf **D** flower **E** calyx **F** open capsule. Scale bar: 20 cm (**A, B**), 1 cm (**C, D, E, F**).

**Figure 3.**
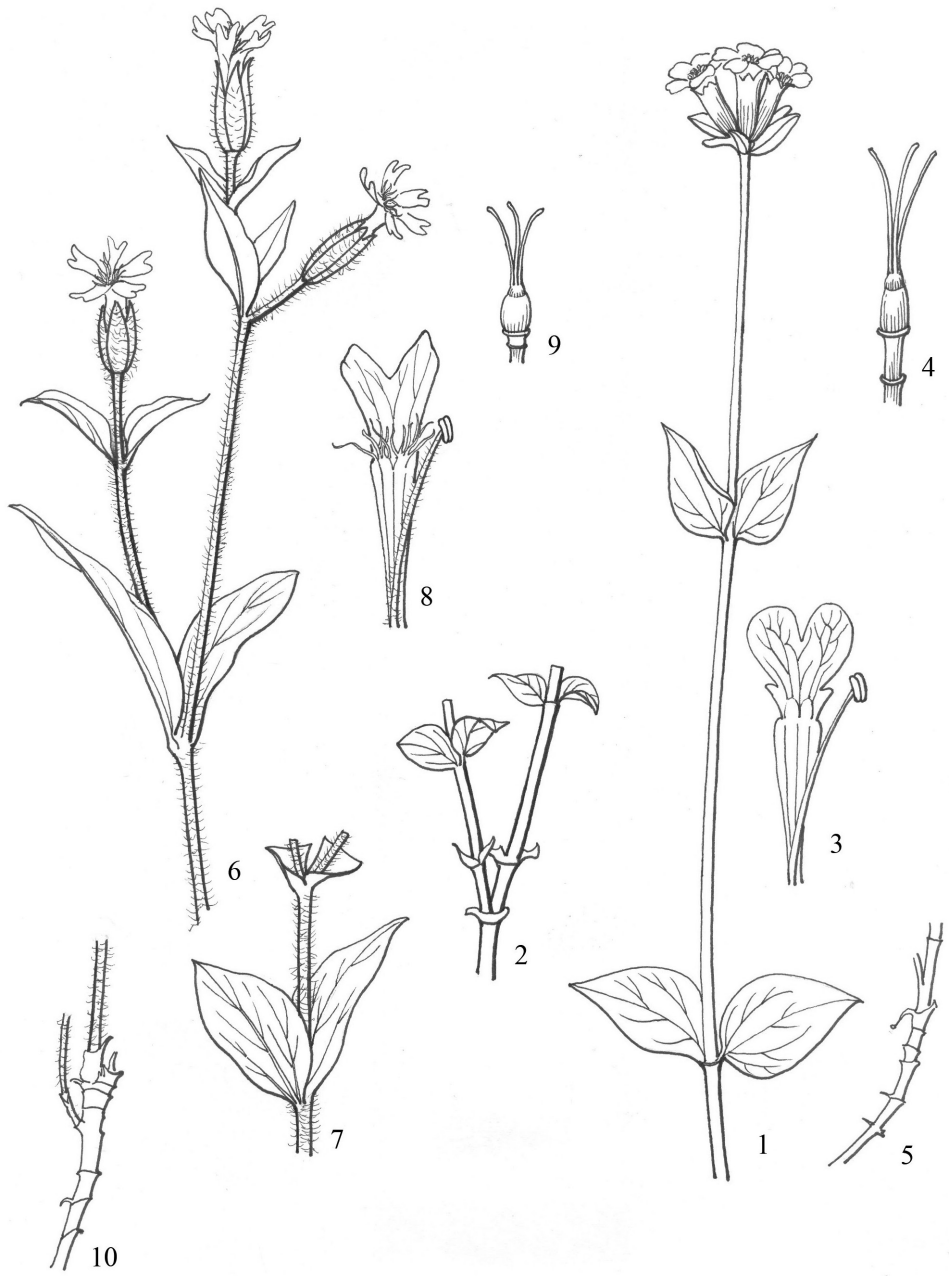
**1–5***Silene platyphylla* Franchet (modified from illustration in flora of China), **6–10***Silene sunhangii*, **1, 6** flowering branch **2, 7** sterile branch **3, 8** petal and stamen **4, 9** pistil **5, 10** root.

Herbs perennial. Plant with densely ciliate, tuberous roots and dichasial cymose inflorescences containing many flowers. Stems diffuse, 30–80 cm tall, much-branched, pubescent. Leaves elliptic, 4–10 × 1–5 cm, glabrous, conspicuously 3-veined. Pedicel 20–30 mm long, pubescent. Calyx tubular-clavate, ca. 1.5–2 cm long, densely hairy on veins; teeth triangulate, ciliate. Petals pale purple to red, ca. 2.5 cm long; claws exserted beyond calyx; catacorolla tasseled, limbs obovate, bifid to 1/3; lobes lanceolate, without a linear lobe or narrow tooth on each side. Stamens slightly exserted; filaments pubescent. Capsule ovoid, 10–20 mm long. Seeds dark brown, reniform, ca. 1 mm long, with lateral auricular pits (Fig. 2, 3).

##### Phenology.

Flowering occurs from February to April, and fruiting from April to June.

##### Etymology.

The new species is named in honor of Chinese botanist, Prof. Hang Sun, who has made significant contributions to the flora of China.

##### Distribution, habitat and conservation status.

*Silene sunhangii* is presently known from only Hubei and Hunan provinces in central China (Fig. 1). It grows in humid and evergreen or deciduous mixed forest, from 1214–2227 m (Fig. 1). A total of three natural populations have been located, each comprising less than 100 individuals distributed over an area not exceeding 100 m^2^. These populations are located within the Shennongjia National Nature Reserve (Hubei province), Houhe Nature Reserve (Hubei Province) and Huping Mountains (Hunan Province), and are therefore well-protected; there are no known threats to these populations. Further field studies are needed to more authoritatively determine the geographic range and frequency of this species. In the meantime, current evidence indicates that *Silene sunhangii* should be assigned the conservation status of “Data Deficient (DD)”, following the IUCN Red List Criteria and Categories (IUCN 2017).

##### Taxonomic notes.

*Silene sunhangii* is a perennial with densely ciliate, tuberous roots and dichasial cymose inflorescences containing many flowers. These characters indicate that the new species should be assigned to Silene sect. Cucubaloideae subsect. Silene Chowdhuri. It can be distinguished from all other species of *Silene* that possess lilac to red petals through its root, stem, leaf and corolla characters as described above. Morphologically, *Silene sunhangii* shows greatest similarities with *S. platyphylla*. The diagnosis above enables the two species to be reliably distinguished. *Silene platyphylla* is distributed in western Yunnan.

## Molecular phylogenetic analysis

**Figure 4.**
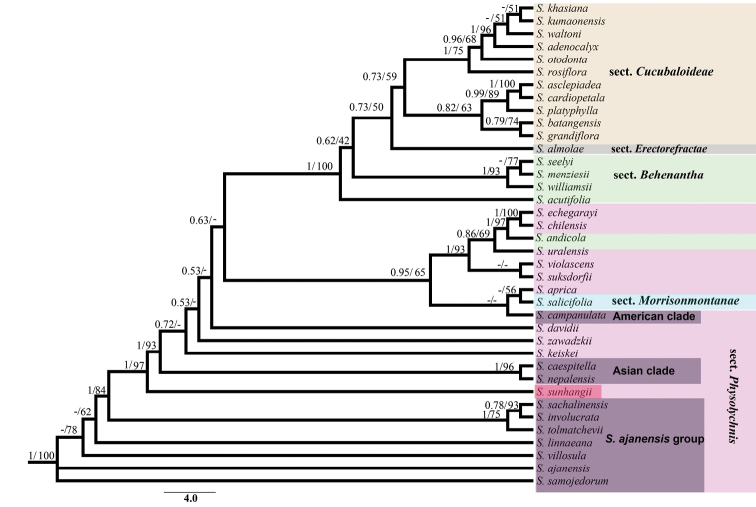
Phylogeny of *Silene* species studied based on ITS region and only bootstrap values >50% are shown. The colored taxa with identical color represent which are from same sect.

The results of our initial phylogenetic analysis, which included over 300 species, are not shown here but they did confirm the position of the new species within *Silene*. In Fig. 4, we present only those clades (38 species from that original matrix) which are relatively close to the new species. Due to the vagueness of outgroup, we constructed unrooted phylogenetic tree based on 38-taxon of *Silene* (Fig. 4). Clades associated with *Silene platyphylla* are also included because morphological criteria indicate that this species has similarities with *S. sunhangii*.

The aligned matrix consisted of 676 characters from 38 species, of which 165 were variable and 82 were parsimony-informative. Our results based on ITS produced trees with identical topology between BI and ML, and only the tree with bootstrap support values from ML analyses was presented (Fig. 4). According to these results, *Silene sunhangii* is nested within a grade that incorporates a polyphyletic Sect. Physolychnis (Benth.) Bocquet. *S. sunhangii* is shown to be separated from associated taxa with very high support (BS = 97, PP = 1), and is well-removed from *S. platyphylla*. These results differ from those of the morphological study which placed *S. sunhangii* in sect. Cucubaloideae and showed it to be morphologically most similar to S. *platyphylla*. As already noted, Sect. Physolychnis was resolved as polyphyletic. This section was shown to include the ‘*S. ajanensis* group’, an Asian clade, an American clade, and miscellaneous other species. These results are consistent with those of a previous study by Petri and Oxelman (2011). An unexplainable result was that *S. platyphylla* was well-separated from *S. sunhangii*, and included within a clade containing species of Sect. Cucubaloideae Edgew. et Hook. f.. These genetic results do clearly support the morphological data in recognizing *Silene sunhangii* as a distinct species. However, relationships of the new species do require further investigation.

## Supplementary Material

XML Treatment for Silene sunhangii

## Appendix 1

**Table:** Vouchers information and GenBank accession of species used in our study.

Species	GenBank accessions	Herbarium voucher specimens
*Silene ajanensis* Vorosch.	KX757376	Anja Rautenberg 68 UPS
*Silene samojedorum* (Sambuk) Oxelman	JX274522	–
*Silene villosula* (Trautv.) V.V.Petrovsky & Elven	KX757382	Afonina et al. 1983. Vii. 23 S
*Silene linnaeana* Vorosch.	KX757383	H. Wilh. Arnell S
*Silene involucrata* (Cham. & Schltdl.) Bocquet	KX757387	Greve Alsosreas Tribsch O
*Silene sachalinensis* F. Schmidt	KX757394	Popov 1949.Vii.8 LE
*Silene tolmatchevii* Bocquet	KX757396	M.Karavaev 1945.Vii.6 LE
*Silene caespitella* F.N. Williams	KX757337	KGB 113 GB
*Silene andicola* Gillies ex Hook. & Arn.	KX757338	–
*Silene violascens* (Tolm.) V.V.Petrovsky & Elven	KX757343	H. Solstad, R. Elven 04/1353 O
*Silene chilens*is (Naudin) Bocquet	KX757359	B. Frajman, P. Schonswetter 12153
*Silene echegarayi* (Hieron.) Bocquet	KX757360	B. Frajman, P. Schonswetter 12176
*Silene zawadzkii* Herbich	KX757363	Cernoch F 47354 M
*Silene davidii* (Franch.) Oxelman & Lidén	KX757367	Frida Eggens 86 UPS
*Silene salicifolia* C.L. Tang	KX757372	Tang 1225 KUN
*Silene nepalensis* Majumdar	JF978562	KIB-D389
*Silene keiskei* Miq.	DQ908643	–
*Silene suksdorfii B.L. Rob.*	DQ908670	–
Silene uralensis subsp. apetala	JX274519	–
*Silene aprica* Turcz. (L.) Bocquet	JF978553	A519
Silene campanulata subsp. glulosa	DQ908635	clone 2459
*Silene adenocalyx* F.N. Williams	KX757269	Poelt J. M
*Silene khasiana Rohrb.*	KX757270	Einarsson et.al 3025 UPS
*Silene waltoni* F.N. Williams	KX757272	G. S. Miehe 03-048-12 Miehe
*Silene kumaonensis* F.N.Williams	KX757273	G. S. Miehe 01-109-08 Miehe
*Silene rosiflora* Kingdon-Ward	KX757277	G. Miehe SonamCo L.Opgenoorth 04-086-01 Miehe
*Silene otodonta* Franch.	KX757282	G.Miehe, U.Wuendisch 94-141-15 Miehe
*Silene asclepiadea* Franch.	KX757283	Boufford D. E. et al. 35267 M
*Silene cardiopeta*la Franch.	KX757284	Liden 4-17
*Silene grandiflora* Franch.	KX757286	KGB 275 GB
*Silene batangensis* H. Limpr.	KX757288	Miehe 07-26-07 Miehe
*Silene williamsii* (Britton) Hultén	KX757298	C. Brochmann H. H Grundt
*Silene acutifolia* Link ex Rohrb.	KX757318	Bengt Oxelman 2554 GB
*Silene almolae* J.Gay	KX757424	Merxmueller H. & Lippert W. 25372 M
*Silene menziesii* Hook.	DQ908651	–
*Silene seelyi* C.V. Morton & J.W. Thomps.	DQ908666	–
*Silene sunhangsii*	–	KUN060722
*Silene platyphylla* Franch.	–	KUN0514438
